# Biomimetic extracellular matrix coatings improve the chronic biocompatibility of microfabricated subdural microelectrode arrays

**DOI:** 10.1371/journal.pone.0206137

**Published:** 2018-11-01

**Authors:** Flavia Vitale, Wendy Shen, Nicolette Driscoll, Justin C. Burrell, Andrew G. Richardson, Oladayo Adewole, Brendan Murphy, Akshay Ananthakrishnan, Hanju Oh, Theodore Wang, Timothy H. Lucas, D. Kacy Cullen, Mark G. Allen, Brian Litt

**Affiliations:** 1 Center for Neuroengineering and Therapeutics, University of Pennsylvania, Philadelphia PA, United States of America; 2 Department of Neurology, University of Pennsylvania, Philadelphia PA, United States of America; 3 Department of Physical Medicine & Rehabilitation, University of Pennsylvania, Philadelphia PA, United States of America; 4 Center for Neurotrauma, Neurodegeneration & Restoration, Corporal Michael J. Crescenz VA Medical Center, Philadelphia PA, United States of America; 5 Department of Mechanical Engineering and Applied Mechanics, University of Pennsylvania, Philadelphia PA, United States of America; 6 Department of Bioengineering, University of Pennsylvania, Philadelphia PA, United States of America; 7 Department of Neurosurgery, University of Pennsylvania, Philadelphia PA, United States of America; 8 Department of Electrical and Systems Engineering, University of Pennsylvania, Philadelphia PA, United States of America; University of Houston, UNITED STATES

## Abstract

Intracranial electrodes are a vital component of implantable neurodevices, both for acute diagnostics and chronic treatment with open and closed-loop neuromodulation. Their performance is hampered by acute implantation trauma and chronic inflammation in response to implanted materials and mechanical mismatch between stiff synthetic electrodes and pulsating, natural soft host neural tissue. Flexible electronics based on thin polymer films patterned with microscale conductive features can help alleviate the mechanically induced trauma; however, this strategy alone does not mitigate inflammation at the device-tissue interface. In this study, we propose a biomimetic approach that integrates microscale extracellular matrix (ECM) coatings on microfabricated flexible subdural microelectrodes. Taking advantage of a high-throughput process employing micro-transfer molding and excimer laser micromachining, we fabricate multi-channel subdural microelectrodes primarily composed of ECM protein material and demonstrate that the electrochemical and mechanical properties match those of standard, uncoated controls. *In vivo* ECoG recordings in rodent brain confirm that the ECM microelectrode coatings and the protein interface do not alter signal fidelity. Astrogliotic, foreign body reaction to ECM coated devices is reduced, compared to uncoated controls, at 7 and 30 days, after subdural implantation in rat somatosensory cortex. We propose microfabricated, flexible, biomimetic electrodes as a new strategy to reduce inflammation at the device-tissue interface and improve the long-term stability of implantable subdural electrodes.

## Introduction

Implantable devices for restoring, replacing or controlling lost or dysfunctional neural circuits are a valuable therapeutic option for a variety of diseases of the central, peripheral, and autonomic nervous systems. Fueled by the miniaturization of electronic and power supply components, as well as by the advances in systems neuroscience [1] a new generation of implantable devices has emerged for mapping cortical circuits [2–4] and implementing neuromodulation-based therapies for Parkinson’s disease [5], epilepsy [6–8], depression [9,10], and mood disorders [11–13]. Research in brain-computer interfaces (BCI) has also led to impressive demonstrations of the potential of cortical neuroprostheses to restore motor and sensory functions in paralyzed patients [14–18]. Implantable electrodes establish intimate contact between man-made devices and neural circuits, and are a core component of all these technologies. Yet, the long-term stability and reliability of electrode implants, especially in the brain, still hampers the clinical translation of many diagnostic and therapeutic neurotechnologies.

Clinical and research intracranial electrodes can be classified into those that penetrate the brain parenchyma and are implanted in cortex or a specific brain structure (a.k.a. “depth” electrodes), and subdural electrodes (a.k.a. ECoG electrodes), typically strips or grids of metal contacts arranged on a polymer substrate that sit on the cortical surface without penetrating it. A large number of studies have investigated the issue of reliability and biocompatibility of penetrating electrodes, especially in the context of intracortical microelectrodes for BMIs. Histological analysis of the foreign body reaction to intracortical microelectrodes implanted in animal models, evidenced the issues of severe inflammation, neurodegeneration and scarring around the electrode implant.

However, studies in human patients focusing on the inflammatory response to subdural electrodes evidenced severe histopathological alterations as early as 1 day after implantation in more than 50% of patients [19]. Furthermore, comparative evaluation in patients simultaneously implanted with depth and subdural electrodes demonstrated that subdural implants elicited a significantly more severe inflammatory reaction than penetrating devices. Finally, longitudinal impedance monitoring in patients chronically implanted with responsive neurostimulators demonstrated that the impedance of the subdural electrodes increased by more than 53% over the course of the first 100 days [20,21], whereas the impedance of depth electrodes in the same subjects only increased by 22% [20,21]. These impedance variations are likely due to the formation of fibrous scar tissue around the implant. As ECoG recordings from chronic subdural electrodes are processed by algorithms in closed-loop neurostimulators, such marked variation in the impedance can strongly impact signal power and quality, thus significantly affecting detection algorithm performance, clinical decision-making [22] and, ultimately, patient outcome. Despite this growing body of evidence of the extensive tissue reaction severely affecting the reliability of subdural electrodes, however, no available study has investigated potential strategies to promote the long-term integration of subdural electrode and mitigate the scar tissue formation.

Studies on intracortical BCI electrodes suggest that, in addition to minimizing implant footprint [23–25] and mechanical stiffness [26–28], the integration of biological material at the electrode-tissue interface can play a major role in determining the extent of the foreign body reaction and fibro-gliotic encapsulation around synthetic implants. Examples of this biomimetic approach [29] include controlled release of curcumin,[30] dexamethasone [31,32] and other anti-inflammatory agents [33], functionalization with neuron adhesion factors [34] and hydrogel coatings [35]. The ECM is a non-cellular scaffold present in all tissues and, in the brain, makes up for approximately 10–20% of the total parenchymal volume. The ECM provides not only structural support to anchor cells, but it also regulates the diverse biochemical cues that guide neurogenesis, neuronal differentiation, survival, axonal growth, pathfinding, and synaptic plasticity [36]. Biomimetic coatings based on passive adsorption of covalent immobilized ECM proteins, such as laminin [37] and fibronectin [38] have been shown to reduce chronic microglia and astrocytic reactivity to silicon and metal intracortical microelectrode implants. In a recent work published by our group [39], we demonstrated that flexible depth microelectrodes coated with collagen and Matrigel films not only mitigate astrogliotic scarring, but also promote neuronal survival compared to stiff silicon implants.

In the present study we evaluate whether a biomimetic approach based on ECM protein coatings, coupled with an ultra-compliant electrode structure, is a viable strategy for mitigating the chronic foreign body reaction to subdural microECoG arrays. To integrate ECM coatings on microfabricated flexible electrodes, we developed a high-throughput batch fabrication process that combines standard photolithographic patterning of microscale metallic features onto flexible polymeric substrates with direct micro-transfer molding and excimer laser micromachining of the ECM films. Using this custom approach, we show the feasibility of producing subdural flexible microECoG arrays predominantly comprised of natural materials and demonstrate that the mechanical, electrical and *in vivo* recording properties are comparable to those of the same flexible arrays without any coating. The naturally occurring ECM is a composite of a collagen base matrix–which constitutes up to 30% of the total protein mass, and provides tensile strength and structural integrity [40]—associated with different fibrous proteins, each providing specific biochemical cues to the cellular environment. In this study we also demonstrate the possibility of assembling freestanding films solely from collagen I and composites of collagen I and fibronectin, and test whether the film composition contributes to the modulation of the astrogliotic response to subdural microECoG implants.

## Materials and methods

### Fabrication of the microECoG electrode arrays

Fabrication of microECoG electrodes began with the fabrication of the thin Au-parylene constructs, which served as controls and as the internal “core” of the ECM electrodes, using standard microfabrication methods previously described by Shen *et al*.[39]. Briefly, a ~ 5 μm-thick layer of Parylene C (poly-monochloro-para-xylylene, Specialty Coating Systems Inc.) was deposited on a silicon carrier wafer by chemical vapor deposition (CVD, PDS 2010, Specialty Coating Systems Inc.). A 100 nm-thick layer of Au was e-beam evaporated on the parylene substrate (Kurt J. Lesker Co.) patterned with photoresists. The 50 μm x 50 μm electrode sites and connecting traces were then defined using a lift-off process in acetone. To encapsulate the gold layer, another layer of parylene C (5 μm in thickness) was deposited via CVD. Subsequently, a patterned layer of Al (e-beam evaporated, 100 nm in thickness) was defined via photolithography and lift-off on the top parylene layer, serving as an etch mask.

The electrode sites and contact pads for interfacing to an external data acquisition system were exposed using reactive ion etching (RIE) of the top parylene layer though the Al etch mask. The Al mask was then removed using wet etching and the Au-parylene constructs were lift-off from the wafer by immersion in DI water. For interfacing with the data acquisition system, the contact pads of microECoG electrodes were bonded to anisotropic conductive film (ACF, Elform Heat Seal Connectors) and connected to a custom-built interface board.

### ECM coating

To fabricate the ECM-electrodes, the Au-parylene constructs were encapsulated with ECM films formed via micro-transfer-molding. Specifically, two types of ECM films were prepared: collagen I film and collagen I/fibronectin. The collagen I solution was composed of Type I rat tail collagen in a 3 mg mL^−1^ solution (Corning, Corning, NY), 10X phosphate buffered saline (PBS), and 0.1 M NaOH at a ratio of 13:2:1 by volume. Fibronectin/collagen I solution was formed by adding fibronectin powder (Sigma Aldrich, St. Louis, MO) to the collagen I solution such that the total protein content comprised 92% Type I collagen and 8% fibronectin by weight. The collagen I solution and the fibronectin/collagen I solution were polymerized at 37°C and 96% humidity for 24 hours to form ECM hydrogels, then dried in air at 37°C for 24 h, followed by rinsing with DI water three times, to form the ECM films.

**Fig 1:**
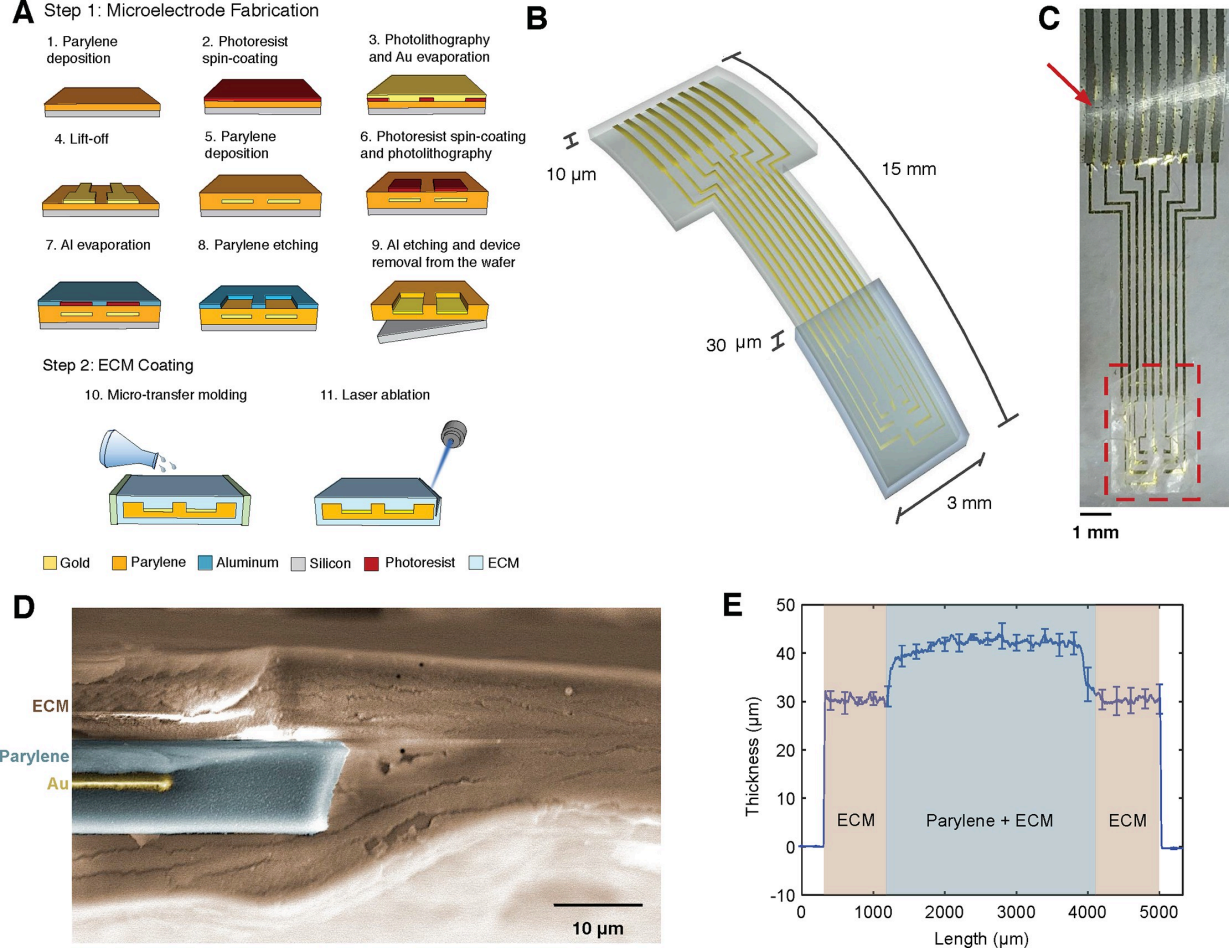
Fabrication of ECM-coated electrode arrays. **(A)** Schematics of the fabrication process of the Au-parylene microECoG arrays (step 1), followed by micro-transfer molding to form the ECM film and UV excimer laser ablation. **(B)** 3D schematics and dimensions of the ECM-coated arrays (thicknesses not drawn to scale). **(C)** Representative electrode array coated with a collagen film (dashed area) and assembled with the ACF connector (arrowhead). **(D)** False colored SEM image of the cross-section of the Au-parylene array coated with the ECM film, post UV excimer laser ablation. **(E)** Thickness profile of the ECM-coated arrays.

The ECM-encapsulated devices were then ablated using a UV excimer laser (193 nm, IPG Microsystems) to conform to the shape of the Au-parylene constructs and then stored covered overnight at ambient conditions. After overnight drying, the thickness of the complete devices was measured with a KLA-Tencor P7 profilometer. The total thickness of the ECM coating (top and bottom layer) was 30.0 ± 1.6 μm (Fig 1E).

### Bending stiffness analysis

Bending stiffness of the uncoated Au microECoG arrays was calculated as:

$$K=E_{p}w_{p}h_{p}^{3}/12$$ Eq 1

where *E*_*p*_ = 2.76 *GPa* is the Young’s modulus of parylene C [41] and *w*_*p*_ and *h*_*p*_ are the width and thickness of the microECoG array. For collagen-coated arrays, the thickness of collagen layer is comparable to that of the parylene encapsulation and, thus, the bending stiffness was calculated with the following modified version of Eq 1 [42,43]:

$$K=E_{c}(wh^{3}-w_{p}h_{p}^{3})/12+E_{p}w_{p}h_{p}^{3}/12$$ Eq 2

where *E*_*c*_ is the Young’s modulus of collagen in the dry or hydrated state [39] and *w* and *h* are the width and thickness of the collagen film, respectively.

### Impedance characterization

Electrochemical impedance spectroscopy (EIS) on bare Au or ECM-coated microECoG electrodes was performed with a Gamry Reference 600 potentiostat (Gamry Instruments) in a phosphate buffered saline bath (PBS) pH 7.4 at room temperature. EIS measurements were acquired by applying a 20 mV rms sinusoidal voltage input in the 1 Hz– 100 kHz range to a three-electrode electrochemical cell, with potentials referenced to Ag/AgCl (Sigma Aldrich), a graphite rod as counter electrode (Bio-Rad Laboratories, Inc.) and an electrode site on the microECoG arrays as the working electrode.

To characterize the electrochemical properties of the Au and ECM interfaces, EIS data were fitted to equivalent circuit models of the interface impedance. Specifically, the interface impedance of the uncoated Au electrodes was modeled with a Randles circuit [44] modified to include the contribution of potential parasitic capacitance arising from the parylene insulation, whereas the ECM-coated electrodes were fitted to a custom defined model to account for the additional interface created by the ECM layer. In the modified Randles cell model for the uncoated Au electrodes the electrode-electrolyte interface is represented by the parallel of the ionic double layer (Zdl) impedance and the charge transfer resistance (Rct), in series with the spreading resistance of the ionic medium (Rs). The double layer impedance is Zdl = [Ydl(jω)^n^]^-1^, where Ydl is the equivalent capacitance, ω the frequency (in radians) and 0<n<1 is a fitting parameter. Zdl represents non-ideal capacitive charge transfer due to electrode surface inhomogeneities (i.e., constant phase element). Rct is a resistive element representing redox reactions involving direct electron transfer through the electrode interface. The pure capacitive element Cp in parallel with the electrode interface accounts for the stray-capacitance of the parylene passivation layer. In the ECM coated electrodes, the additional impedance of the protein layers is modeled by a parallel of capacitive and resistive elements, respectively Zcoat and Rcoat [45,46].

Optimal values of model parameters were found by fitting the EIS data to the equivalent circuit models with a modified version of the Matlab code Zfit [47] and the Gamry Echem Analyst fitting tool (Gamry Instruments).

The theoretical value of the spreading resistance in the electrolyte was calculated from the following expression for rectangular electrodes [48]:

$$R_{s}=\rho \frac{ln(4l/w)}{\pi l}$$ Eq 3

where *⍴* = 72 Ωcm is the resistivity of PBS at 25°C, *l* and *w* are the length and the width of the electrode contact sites, respectively.

The value for the parasitic capacitance through the parylene passivation layer was obtained by measuring the EIS of parylene in the frequency range of 5 kHz– 1 MHz in PBS with the same experimental setup described above (i.e., sinusoidal voltage input 20 mV rms, three-electrode configuration). Below 5 kHz the noise in the system made impossible to obtain any reliable measure. From the EIS spectra, the value of the parasitic capacitance C_pcalc_ was calculated as:

$$C_{pcalc}={\left(2\pi fZ_{p}\right)}^{-1}=6.5\pm 3.25\;pF$$ Eq 4

where *f* is the frequency in Hz and *Z*_*p*_ is the modulus of the impedance measured with EIS.

### Animal surgeries

For acute neural recordings one 330 g Sprague-Dawley rat was anesthetized with an intraperitoneal injection of ketamine (60 mg/kg) and dexmedetomidine (0.25 mg/kg) and placed in a stereotaxic frame. A craniotomy was performed to expose the right barrel cortex and the dura was removed. A skull screw was placed in the left parietal bone to serve as the reference electrode for the recordings. The microECoG arrays (uncoated or with ECM coatings) were placed over the exposed cortical surface. ECoG signals from the 8 recording sites simultaneously were acquired with a commercial electrophysiology system (Tucker-Davis Technologies) in epochs of 5 min duration for each electrode type.

For histological evaluation, the Au-parylene constructs were first sterilized using autoclaving treatment at 121°C for 20 min prior to ECM coating. Then, the ECM-coated and the Au-parylene electrodes were exposed to UV radiation, for 2 hours, in a sterile biological safety cabinet and stored in sterile packaging until used. Male Sprague-Dawley rats (325-350g, n = 4 for each time point across all three groups, total n = 24) were anesthetized with isoflurane and mounted in a stereotactic frame. The scalp was cleaned with betadine, bupivacaine was injected along the incision line, and a midline incision was made to expose the Bregma landmark. A 3 x 3 mm craniotomy was performed at the following coordinates relative to Bregma: -2.3 mm (AP), -3.5 mm (ML). The dura was opened and the microECoG array, (uncoated or with ECM coatings) was carefully placed on the surface of the brain. The perimeter of the microECoG array was placed under the surrounding dura to minimize any potential any micromotion or mismatched stiffness that might result in an elevated immunological response. To avoid the potential influence of implantation and fixation procedures that have been suggested to exacerbate the inflammatory reaction, such as tethering the device to the skull [24,49,50], only the distal portion of the arrays highlighted in Fig 1C was implanted subdurally. The scalp was sutured closed and buprenorphine was given for postoperative analgesia. Animals were transferred to a warming pad to recover from anesthesia, and were then pair-housed for the remainder of the study.

All animals used for this study where purchased from Charles River Laboratories, Inc. and pair-housed in microisolation cages within a dedicated ABSL-1 facility. The animals were daily monitored by trained animal technicians and lab personnel. Excluding euthanasia, no animal died due to experimental procedures. All procedures were approved by the Institutional Animal Care and Use Committees at the University of Pennsylvania and the Michael J. Crescenz Veterans Affairs Medical Center and adhered to the guidelines set forth in the NIH Public Health Service Policy on Humane Care and Use of Laboratory Animals (2015).

### Analysis of *in vivo* neural recordings

Power spectral densities were estimated by computing the discrete Fourier transform for each 5 second Hamming-windowed segment and averaging across the entire ECoG recording. Cycle-triggered wavelet scalograms were computed by first identifying the peak of each cycle of the ~1Hz slow oscillation from the instantaneous phase of the narrow-band (0.5–2 Hz) Hilbert-transformed signal. Second, the continuous wavelet transform (CWT) was applied to the wide-band signal in a window around each cycle peak, using the complex Morlet wavelet with a bandwidth and center frequency of 1 Hz and scales corresponding to pseudo-frequencies logarithmically spaced from 3 to 500 Hz. Third, the 5% trimmed mean of the magnitude of the CWT coefficients across cycles was computed. Finally, the magnitude in a -0.5 to 0.5 s window was expressed as a percentage of the mean magnitude in a baseline window (-1.3 to -1 s window prior to the cycle peaks) in which the average narrowband (0.5–2 Hz) signal was unmodulated.

### Chronic histology

At 7 or 30 days post-surgery the animals were anesthetized and underwent transcardial perfusion with heparinized saline followed by 10% paraformaldehyde (PFA). After extracting the brain and removing the electrode the tissue was stored in 30% sucrose for cryopreservation. Next, the tissues were mounted in OCT (Tissue Tek–Fisher Scientific supplier) and frozen in dry ice/2-methylbutanol. Tissue was then sliced coronally into sections of 20 μm with a microtome. Sections were rinsed in 1x PBS, and then permeabilized at room temperature using 0.3% Triton-X100 plus 4% normal horse serum (Vector Labs) for 60 minutes. Primary antibodies diluted in 1x Optimax (Biogenex) with 1% normal horse serum were applied and allowed to incubate overnight at 4°C. Sections were stained with anti-rabbit IBA1 (1:1000; Wako) and anti-goat-GFAP (1:1000; Abcam). Secondary antibodies were applied at room temperature (donkey-anti-rabbit 568 (1:1000; Life Technologies) and donkey-anti-goat 647 (1:1000; Fisher). Sections were then quickly rinsed, incubated with hoechst 33342 (1:10,000) for 10 minutes, and then mounted on glass slides with coverslips using Fluoromount-G mounting media (Fisher). Glial reactivity was quantified in an automated fashion by measuring the pixel intensity of the ipsilateral cortex ROI normalized to the contralateral cortical ROI using NIS Elements (10x objective, 1024x1024, Nikon Instruments, Japan; S1 Fig). Background fluorescence was subtracted using a small ROI in the contralateral corpus callosum without any reactive cells. Mean intensity values were obtained by averaging the individual intensity values at each level across all animals. For all experiments, data are presented as mean ± standard error of the mean. Tests for significant differences between groups and the uncoated controls were performed using one-sampled t-tests. Two-sided analysis was performed for each test with a Type I error rate of 0.05 using GraphPad Prism version 6.0 (GraphPad software, CA, USA).

To quantify the number of microglia/macrophages, representative regions from the ipsilateral cortices below the arrays were imaged (20x objective, 1024x1024 pixels, 1100 μm x 881) and Iba1+ cells were manually quantified. Results were compared with one-way ANOVA. For all experiments, data are presented as mean ± standard error of the mean.

## Results

### Electrode fabrication

Fig 1 shows the custom fabrication process developed for assembling ECM films on soft electrode structures and characterization of the microECoG electrode arrays. The microfabrication process consists of a standard sequence of photolithography, metal lift-off and etching steps (Fig 1A). The final layout of our microECoG devices is a 3 mm x 15 mm (W x L) 8-channel array with 50 μm x 50 μm gold recording sites, arranged around a 570 μm x 570 μm central window and spaced 260 μm apart (Fig 1B and 1C). To deposit ECM films on the distal end of the arrays, randomly selected Au-parylene devices are lifted-off from the silicon carrier and placed in a polydimethylsiloxane (PDMS) mold, where a collagen or collagen-fibronectin precursor solution is casted and polymerized in a controlled humidity and temperature environment. After polymerization, the final layout of the device is precisely defined by ablating the excess protein film via UV excimer laser micromachining, which excites and dissociates the molecular bonds while preventing excessive heating and decomposition to elemental compounds that would result in protein denaturation [51]. Imaging of the device cross section after UV ablation (Fig 1D) confirms the integrity of the protein structure and shows that more than 60% of the implanted area of the arrays is composed of natural material, with the ECM film forming a 20 μm thick layer completely encapsulating the Au-parylene synthetic interface.

### Mechanical characterization

The bending stiffness of an electrode implant is one of the main factors affecting the extent and severity of the chronic inflammatory response. The stiffer the electrode the more intense will be the frictional forces at the electrode-tissue interfaces arising from the brain micromotion, which lead to tissue deformation, vascular damage and sustained inflammation [26,27]. The bending stiffness of a neural electrode is strongly controlled by its thickness [52] via a third power dependence, and by the elastic modulus of the constituting materials [53]. The bending stiffness of our 10 μm-thick uncoated Au-parylene microECoG arrays is kb_Uncoated_ = 6.9 x 10^−10^ Nm^2^, which is among the lowest values reported for neural microelectrodes [43,54]. The presence of the ECM coatings results in a 4-fold increase in the final thickness of the arrays. However, the collagen films hydrate upon exposure to an aqueous environment, such as the physiological cerebrospinal fluid and the saline solution flushed intraoperatively, leading to a drop in the film elastic modulus from 3.4 GPa in the dry state to 2.6 MPa in the hydrated condition [39]. Thus, in the hydrated state the bending stiffness is kb_ECMwet_ = 7.3 x10^-10^ Nm^2^, which shows that presence of ECM coatings does not affect the bending stiffness of the implanted devices.

### Electrochemical impedance characterization

**Fig 2:**
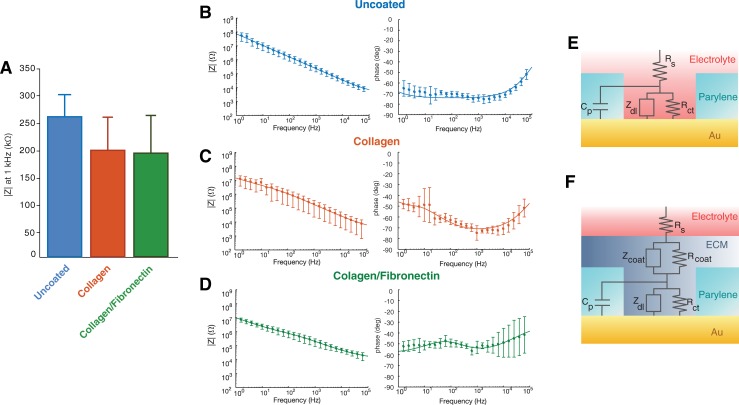
Impedance characterization. **(A)** Impedance modulus at 1 kHz of the uncoated, collagen collagen-fibronectin coated arrays. **(B-D)** Magnitude and phase spectra of the impedance measured *in vitro* of the Au-parylene electrode arrays **(B)** uncoated or coated with 20 μm film of **(C)** collagen, **(D)** collagen-fibronectin (n = 8). Overlaid lines are the impedance modulus and phase calculated from fitting the experimental data with equivalent circuit models in (E) and (F). **(E, F)** Equivalent circuit of the electrode/electrolyte interface of the **(E)** uncoated Au-parylene and **(F)** ECM coated arrays.

Neural electrodes function by detecting changes in the extracellular field generated by ionic currents flowing in the local microenvironment. The impedance of a microelectrode represents the frequency-dependent resistance offered to this flow of ions by the electrode-extracellular medium interface and strongly affects the spatiotemporal resolution of the recorded signals, as well as the noise characteristics of the electrodes [55]. We tested the effects of the ECM coatings on the electrochemical impedance of the microECoG arrays with electrochemical impedance spectroscopy (EIS, Fig 2A) and found a small decrease, although not significant, in the impedance modulus at 1 kHz–the characteristic frequency of neural action potentials typically used as a reference for impedance comparison—from the uncoated Au arrays (256 ± 36 kΩ) compared to the same devices coated with collagen (195 ± 61 kΩ) or collagen-fibronectin (190 ± 72 kΩ, p = 0.54). This lack of significant alteration in the electrochemical impedance has been traditionally attributed to the swelling of the protein film, which facilitates hydration and flow of ions to the electrode interface [38,56]. However, the EIS spectra in Fig 2B–2D suggest a more complex contribution of the ECM coatings to the final impedance properties of the electrodes. In the 1-10Hz range the impedance of the ECM coated electrodes is almost 10x lower than in the uncoated state. Furthermore, the phase diagrams show that the phase of uncoated Au electrodes is substantially capacitive with a constant value of ~-70° in the whole 1 Hz– 10 kHz range (Fig 2B), while the phase of the ECM-coated electrodes is closer to -50° in the low-frequency range (Fig 2C and 2D).

To better characterize the electrochemical properties of the microECoG electrodes and understand the effects of ECM coatings on the final impedance properties of the electrode interface, we fitted the EIS spectra to equivalent circuit models of the electrode-electrolyte impedance (Fig 2E and 2F). Table 1 shows the value of the parameters derived from fitting and the values of the Rs and Cp calculated or measured experimentally. The chosen equivalent circuit models appear to represent the electrochemical interface well, given the good agreement between experimental and estimated impedance data in Fig 2B, 2C and 2D, and between the theoretical and fitted values of the circuit parameters. Uncoated Au electrodes behave, as expected, as almost ideally polarizable electrodes: the charge exchange at the electrode interface is dominated by capacitive phenomena and almost no direct electron transfer occurs (Rct Au = 454 MΩ, Ydl Au = 2.7 nS s^n^). The electrodes coated with ECM films show ~10 fold increase in the equivalent capacitance compared to the Au interface (Ydl collagen = 15.5 nS s^n^, Ydl collagen-fibronectin = 28.6 nS s^n^), ~100x smaller charge transfer resistance at the protein interface (Rcoat collagen = 3.9 MΩ, Rcoat collagen-fibronectin = 3.6 MΩ) compared to the Rct of gold and larger values of the capacitive constant phase element Ycoat (Ycoat = 9.3 nS s^n^ for collagen and 55 nS s^n^ for collagen-fibronectin).

**Table 1:** Circuit model parameters obtained by fitting EIS data for to the equivalent circuit models depicted in Fig 2A and 2B.

Electrode type	Fitted								Calculated		
	R_s_ (kΩ)	R_ct_ (kΩ)	Y_dl_ (10^−9^ S s^n^)	n_dl_	R_coat_ (kΩ)	Y_coat_ (10^−9^ S s^n^)	n_coat_	C_p_ (pF)	R_scalc_ (kΩ)		C_pcalc_ (pF)
**Au**	3.4	4.5x10^5^	2.7	0.84	-	-	-	1.5	6.4		6.5
**Collagen I**	2.2	5.1x10^4^	15.5	0.78	3.9x10^3^	9.3	0.8	5.8	6.4		6.5
**Collagen I-Fibronectin**	3.9	1.1x10^5^	28.6	0.79	3.6x10^3^	55	0.5	1.3	6.4		6.5

### Neural recordings *in vivo*

**Fig 3:**
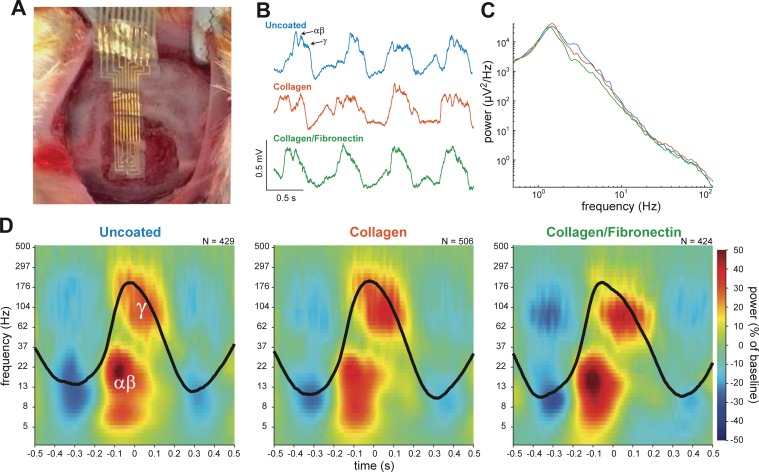
*In vivo* acute recordings of cortical potentials. **(A)** Photograph of the electrode array placed on the surface of the rat barrel cortex. **(B)** Representative 2 s segments of data recorded from the gold (blue trace), collagen-coated (orange trace), and collagen-fibronectin-coated (green trace) electrodes. Note the large amplitude, ~1-Hz oscillations and smaller, faster rhythms occurring near the peak of each cycle (arrows). **(C)** Average power spectral density calculated over a 1 min time window for the three electrode types. **D**, Average cycle-triggered wavelet scalograms for the three electrode types. The color indicates power relative to the phase of the 1-Hz cycle (average cycle shown in black). The number of cycles used to generate the averages is indicated. Power in two frequency ranges, αβ = 5–30 Hz and γ = 50–300 Hz, was coupled to two distinct phases, upslope and peak, of the 1-Hz cycle.

To verify whether comparable impedance characteristics *in vitro* translated into analogous quality and information content of the neural signals acquired *in vivo*, we recorded ECoG activity in an anesthetized adult rat. A 3 mm x 3 mm craniotomy exposed the cortical somatosensory area of the rat under ketamine-dexmedetomidine anesthesia (Fig 3A). Uncoated Au, collagen or collagen-fibronectin electrodes were placed sequentially over the exposed cortical area, connected to the same channels on the amplifier–to avoid channel-related variations in the recorded signal–and used to sequentially acquire ECoG signal in epochs of ~5 min. Fig 3B shows representative 2 s traces of the ECoG recordings acquired with the Au or ECM coated electrodes. For all the three type of electrodes the recorded signal presents up-down-state oscillations of comparable amplitude at 0.5–2 Hz, with fast oscillations in the gamma band (30–90 Hz) appearing during the up states. Analysis of the power spectra of the recordings does not evidence any discernible difference or attenuation in any frequency band, comparing uncoated and ECM-coated electrodes (Fig 3C). The ~1 Hz up-down dynamic is characteristic of ketamine-dexmedetomidine anesthesia, which induces a sleep-like slow rhythm in the rat cortex arising from synchronized states of neuronal excitation (up) and inhibition (down) [57]. Increased excitability in the up state is manifest in the ECoG signal by high-frequency power, either due to asynchronous spiking activity or synchronous activity in defined frequency bands (e.g. the gamma band) [58]. Here, we observed an elevation of gamma band power near the peak of the slow cycle. On the upslope of the cycle, power was elevated in a distinct lower frequency range, near the alpha and beta bands (Fig 3B and 3D). This intimate cross-frequency relationship between the phase of the slow rhythm and power at two different higher frequency bands was detected on all three electrode types (Fig 3D). Finally, the noise floor of the recordings was 6.7 ± 2.4 μV_pp_ for the uncoated Au, 6.4 ± 1.7 μV_pp_ for the collagen and 6.9 ± 1.9 μV_pp_ for collagen-fibronectin coated electrode arrays, thus confirming that the ECM coatings do not adversely affect the impedance properties, and thus, the noise characteristics of the electrodes when used to detect and sort neural signals *in vivo*.

### Chronic inflammatory response

From the mechanical, electrochemical and recording performance, the ECM-based electrodes are comparable to the completely synthetic ones, but does the biological interface reduce the tissue reaction to the foreign material implants, even in the case of an ultra-compliant device?

**Fig 4:**
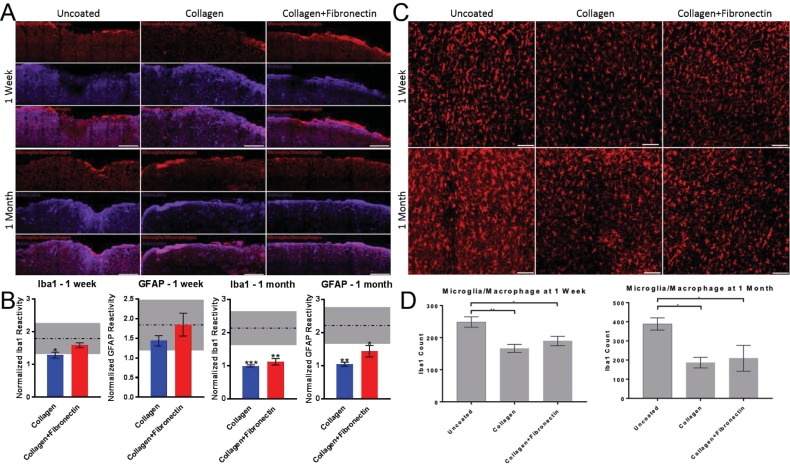
Comparison of Glial Reactivity in Cortex Below Electrode Arrays with and without ECM Coatings. (A) Cortical sections labeled for microglia/macrophages (Iba1, top rows) or astrocytes (GFAP, middle rows) at 7 or 30 days post-implantation. Bottom rows are the overlay of the sections. (B) Mean cortical reactivity from ECM coated arrays (normalized to the contralateral region). Dashed lines represent the reactivity generated from uncoated array implantation. Data is presented as the mean ± standard error of the mean, n = 4 for each electrode type at each time point. * denotes p<0.05, ** denotes p<0.01, and *** denotes p<0.001. Scale bar = 500 μm. (C) Mean Iba1+ cell counts were obtained by quantifying positive cells from cortical sections labeled for microglia/macrophages at 7 or 30 days post-implantation. (D) Fewer Iba1 positive cells were found in cortex below electrodes coated in collagen or collagen-fibronectin than the uncoated control electrodes. Data is presented as mean ± standard error of the mean, n = 4 for each electrode type at each time point. Data was compared with one-way ANOVA. * denotes p<0.05. Scale bar = 100 μm.

To answer this question, we assessed the foreign body reaction to Au-parylene subdural microECoG arrays and compared it to that of the same arrays coated with 30 μm of collagen or collagen-fibronectin. Arrays were implanted subdurally in the rat somatosensory cortex (n = 4 for each type of electrode tested). After a period of 7 or 30 days post-implantation, animals were sacrificed and cortical sections were stained with microglial/macrophage Iba1 or astrocytic GFAP markers, to assess the central nervous system specific inflammatory response. Immunohistochemical analysis of the stained tissue (Fig 4A and 4B) shows that the mean Iba1 pixel intensity at 1 week for the uncoated electrodes was significantly higher than for the collagen coated electrodes (1.8±0.9 *vs*. 1.3±0.2). We did not find statistical significance for Iba1 pixel intensity at 1 week between the uncoated electrodes and collagen-fibronectin coated electrodes (1.6±0.1). We also did not find statistical significance for mean GFAP pixel intensity at 1 week between uncoated electrodes and collagen (1.8 ± 1.3 *vs*. 1.4±0.3) or collagen-fibronectin (1.8±0.6).

However, after 1 month we found a higher mean pixel intensity for the uncoated Au electrodes compared to collagen and collagen-fibronectin coated implants, both for Iba1 (Au: 2.1±1.0, collagen: 1.0 0.1, collagen-fibronectin 1.1±0.12) and for GFAP (Au: 2.2±1.1, collagen: 1.0±0.1, collagen-fibronectin, 1.4±0.3).

Then, we counted the number of microglia/macrophages in the cortex directly below the array (1100 μm x 882 μm, Fig 4C and 4D) and found that already after 7 days post-implant a larger number of Iba1 positive cells had accumulated around the uncoated Au electrodes (248.8±32.4) than around the collagen (166.5±26.64) and collagen-fibronectin coated electrodes (189.5±29.1). After 1 moth post-implant, the number of Iba1 positive cells was still higher in the cortex under the uncoated Au arrays (388.8±63.77) than under the collagen (186.7±48.26) and collagen-fibronectin coated electrodes (209.3±117.5).

We also imaged the explanted microECoG arrays with phase and confocal microscopy after immunolabeling for Iba1 (Methods in S1 Text and S2 Fig). Findings from the phase microscopy were consistent with the preservation of the ECM coatings at 7 days post-implant. At 30 days, we still found evidence of the presence of the coatings, although they appear to have been partially degraded (most likely through enzymatic digestion). Confocal imaging showed accumulation of microglia/macrophages around the all the implants.

When we stained the tissue for neuronal markers neuronal nuclei, NeuN, and dendrites, MAP2), we did not observe any appreciable changes in neuronal density compared to contralateral cortex for any of the treatment groups (data not shown).

## Discussion

Improving the functional longevity and reliability of implanted brain electrodes is a significant unmet need. Implanted arrays for brain-computer interfaces steadily lose fidelity over time because tissue trauma, encapsulation and the brain’s native foreign body response to current implanted materials. In the realm of clinical implants, performance can take up 100 days to stabilize due to these processes, and chronic inflammation degrades electrode impedance, the ability to record brain signals, transfer charge, and overall performance may worsen slowly over time. Improved materials for intracranial implants would be a major contribution to current and next generation research, diagnostic and therapeutic neuro devices. More specifically, in the scope of the current study, the extensive foreign body reaction to subdural electrodes used in pre-surgical seizure mapping and responsive neurostimulation devices critically affects the implant reliability and the efficacy of clinical interventions. Here we sought to evaluate whether addressing the material compatibility issue between the host neural tissue and the implantable subdural arrays could be a viable option to improve the device long-term reliability and reduce the brain’s inflammatory foreign body reaction. Specifically, we proposed a high-throughput, scalable approach for fabricating biomimetic, flexible subdural electrodes based on microscale ECM coatings, and tested it *in vitro* and *in vivo*. The *ad hoc* microfabrication process presented in this work enables high-throughput fabrication of thin film, multichannel electrode arrays and precise patterning of microscale-thin ECM composites within the electrode structure, while preserving the molecular and structural integrity of the constituting proteins. As a demonstration of the feasibility of our approach, we tested two different ECM film compositions, collagen I and collagen I/fibronectin and demonstrated the fabrication of devices composed for more than 60% by natural material. Despite the ~4-fold increase in the final thickness, the mechanical stiffness of the subdural microECoG arrays in the hydrated state is comparable to that of thinner, uncoated arrays, thanks to the ability of the ECM films to transition to a soft hydrogel upon exposure to the aqueous environment of the intracranial space.

EIS measurements revealed no significant difference in the impedance at the reference frequency of 1 kHz after adding the ECM coatings. Fitting the EIS data with equivalent circuit models of the electrode interface, however, revealed a complex contribution of the protein interface to the impedance characteristics in the broad frequency range of 1 Hz-100 kHz, possibly involving enhanced charge accumulation at the electrode-electrolyte interface. Fibronectin and collagen, in fact, have acidic isoelectric points of 5.5 and 4.5 respectively, making the net charge of the molecule negative at the neutral pH of the PBS, which, in turn causes a local increase in the concentration of ions at the protein interface [59,60]. Thus, results from impedance modeling suggest that the ECM layers, not only do not passively hinder flow of ions to the gold interface, but actually might actively contribute to effectively increase the charge distribution at the electrode-electrolyte interface through mixed resistive and capacitive effects. As the characteristic bandwidth of ECoG signals is in the 1–300 Hz and more recent work demonstrated the possibility of even detecting events in the spike-band range (~ 1 kHz) [2], modeling the interface impedance are important to understand and predict the recording properties of ECM coated devices in a broadband frequency range.

Recordings of ECoG signals *in vivo* confirmed that the recording and noise properties of the electrodes are not affected by the ECM coatings and that biomimetic electrodes can adequately resolve both low and high-frequency cortical oscillations characteristic of the anesthetized state. *In vivo* analysis of the temporal progression of the inflammatory reaction to subdural ECM coated arrays revealed a mitigated response of the brain tissue to the implants, with a particularly marked reduction in the inflammatory response at chronic time scales.

The extensive inflammatory reaction to subdural electrodes found in the immediate days to one month following implantation is induced by the violation of the arachnoid space from the dura removal and electrode placement, which can severely damage the cortical blood vessels. The cerebral and the arachnopial surface, in fact, are highly vascularized [61][62] and the hemorrhage from arteriole and vessels triggers the cascade of immune reaction. This reaction, which was found to be moderate to severe in 73% of patients implanted with subdural electrodes, includes infiltration of immune cells in the subarachnoid space and extensive astrogliosis that can last for more than 45 days post-implantation [62][63]. In our study we found that already at 7 days a higher number of microglia/macrophages had migrated at the interface between the tissue and the uncoated electrodes than under ECM-coated implants (area of 1100 μm x 882 μm directly below the electrode implants). At this time point, cortival reactivity for both GFAP and Iba1 appeared to be homogeneously distributed. At 30 days post-implant, the presence of ECM coatings not only significantly mitigated the accumulation of microglia/macrophages at the electrode/tissue interface, but also significantly reduced both microglial and astrocyte reactivity in the entire cortical area, almost to the level of non-implanted control regions. These observations are in agreement with the extent and temporal scales of the response to intracortical microelectrodes coated with nanostructured laminin reported by He *et al*. [37]. Since the microECoG electrode size, shape, materials and fabrication process were exactly the same and we avoided potential additional causes of inflammation, such as tethering the devices to the skull [24,49,50], the improvement in the chronic foreign body reaction in our study can be attributed to the ECM coatings. In this study, a large 3 mm x 3 mm craniotomy was made to allow for placement of the microEcoG array on the surface of the brain similar to previous publications [4,63]. The large craniotomy would prevent bone ossification that might adhere to the electrode leading to micromotion that can cause an elevated chronic response.

A possible mechanism for the observed bimodal response in inflammatory cell reactivity may be that ECM proteins do not suppress, but actually stimulate the acute inflammatory response by promoting microglial activation, upregulation and release of inflammatory cytokines, which further promote the activation and recruitment of proximal microglia and astrocytes [37,64]. This enhanced astrogliotic response in the acute phase might contribute to accelerate the healing process by a coordinated clean-up of the necrotic cellular debris within the first week after implantation. This response appears to be indistinguishable from the reaction elicited by the uncoated synthetic devices. After 30 days, however, uncoated implants still elicit sustained microglial and astrocyte reactivity, while for ECM-coated devices the inflammatory reaction appears to be concluded and the reactivity of both microglia and astrocytes returned to values comparable to that of uninjured tissue. Our results appear to be consistent for both collagen and collagen-fibronectin coated implants at this latter time point, and we have not found any significant effect from the presence of fibronectin on acute and chronic microglia and astrocyte reactivity. Thus, the present study suggests that the sole presence of the collagen interface might be sufficient to mitigate the astrogliotic response [65]. As the formation of a dense sheath of reactive inflammatory cells can severely impact electrode impedance by creating an additional resistive impedance to ion flow [66,67], our overall findings support the hypothesis that a biomimetic approach coupled to electrode flexibility might be a viable option to improve long-term reliability of subdural electrode implants.

## Conclusions

In this study we fabricate and characterize flexible, biomimetic microECoG arrays coated with ECM proteins, and compare their performance to flexible, uncoated devices. In our experiments, the presence of the ECM coatings does not affect mechanical, electrochemical and *in vivo* recording properties of the arrays, but appears to cause a remarkable reduction in the chronic foreign body response compared to uncoated controls. This result highlights the need for designing new devices capable not only of matching the mechanical compliance of brain tissue, but also recapitulating its native material properties and either native or neutral immunoreactivity.

Future work will be devoted to longitudinally investigating how attenuation in the inflammatory reaction to implanted electrodes correlates with chronic recording performance *in vivo*. Although we did not find any significant effect from adding fibronectin to the ECM composite, we plan to test newer coatings that integrate other fibrous ECM proteins, proteoglycans and loading anti-inflammatory agents in the collagen I base matrix. Another planned set of experiments will explore whether a complex biomimetic interface that more closely resembles the composition of the naturally occurring ECM will further improve the biocompatibility and stability of the electrodes. In these same experiments we plan to will explore how to tune ECM composition to modulate the complex cellular mechanisms that guide migration, adhesion and selective activation of specific cell phenotypes.

The microfabrication and characterization methodologies developed in this work can be translated to and open new opportunities for other type of intracranial implants, including penetrating electrodes for deep brain stimulation, BCIs, other types of chronic central neuromodulation as well as for implantable interfaces for peripheral nerve recording, stimulation and regeneration. It is our hope that newer, biomimetic electrode and coating materials will advance and improve the efficacy of implantable diagnostic, therapeutic and research devices for human use.

## Supporting information

S1 Text Methods for the analysis of the explanted microECoG arrays. (DOCX)

S1 Fig Histological evaluation of the inflammatory response. (A) After perfusion, the microECoG arrays were carefully removed and brains were divided into three tissue blocks containing the regions contacting the micro-ECoG array. Blocks were cryoprotected in 30% sucrose and frozen. Sections were then serially cut 20 μm thick from the three levels and stained for Iba1 (microglia/macrophages) and GFAP (astrocytes). (B) Glial reactivity was quantified in an automated fashion by measuring the pixel intensity of the ipsilateral cortex ROI normalized to the contralateral cortical ROI (10x objective, 1024x1024). (C) To quantify the number of microglia/macrophage, representative regions from the ipsilateral cortices below the arrays were imaged with a 20x objective (area: 1024x1024 pixels, 1100 μm x 881 μm), and Iba1+ cells were manually counted. Scale bar: 100 μm.
(TIFF)

S2 Fig Imaging of the explanted microECoG arrays. Representative phase images (A-C, G-I) and multiphoton reconstructions (D-F, J-L) of uncoated (A/D, G/J), collagen-coated (B/E, H/K), and fibronectin-coated (C/F, I/L) microECoG arrays at 1 week (A-F) and 1 month (G-L) post-implant. Arrays were immunolabeled for IBA-1 to identify activated microglia/macrophages.
Scale bars: 500 μm.
(TIFF)
