# Supplementary material for: Biomimetic extracellular matrix coatings improve the chronic biocompatibility of microfabricated subdural microelectrode arrays
Source: PLoS One. 2018 Nov 1;13(11):e0206137. doi: 10.1371/journal.pone.0206137 (PMC6211660; doi:10.1371/journal.pone.0206137)
Supplement: S1 Text — (DOCX) [file pone.0206137.s001.docx]

**S1 Text. Methods for the analysis of the explanted microECoG arrays**

Post-perfusion, microECoG arrays were carefully removed from the fixed brain to assess the presence of residual ECM on the coated arrays and the immune response across uncoated/coated arrays. Phase contrast images were taken on a Nikon Eclipse Ti-S microscope using a QIClick camera and NIS Elements BR 4.13.00 (Nikon Instruments). ECM residues were manually identified on the coated arrays. To assess the immune response, arrays were rinsed in 1x PBS before being immersed in 0.4% Triton + 4% normal horse serum (Vector Labs) in 1x PBS for 60 minutes. Arrays were then rinsed in 1x PBS before being incubated in primary antibody solution (4% horse serum in 1x PBS) containing anti-goat IBA-1 (1:500, Abcam) at 4 ˚C overnight. Following primary antibody incubation, arrays were immersed in secondary

antibody solution (4% horse serum in 1x PBS) containing donkey anti-goat 647 (1:500, Invitrogen) for 2 hours. Arrays were then immersed in Hoechst 33342 (1:10,000) for 10 minutes before being rinsed in 1x PBS and mounted on glass slides for imaging. Immunolabeled arrays were imaged on a Nikon A1RMP+ multiphoton confocal microscope paired with NIS Elements AR 4.60.00, with representative images taken from maximum intensity z-projections of 5-6 µm per z-slice. Qualitative comparisons of immune response were based on signal intensity and morphology.
