# Supplementary figures and images for: Biomimetic extracellular matrix coatings improve the chronic biocompatibility of microfabricated subdural microelectrode arrays
Source: PLoS One. 2018 Nov 1;13(11):e0206137. doi: 10.1371/journal.pone.0206137 (PMC6211660; doi:10.1371/journal.pone.0206137)

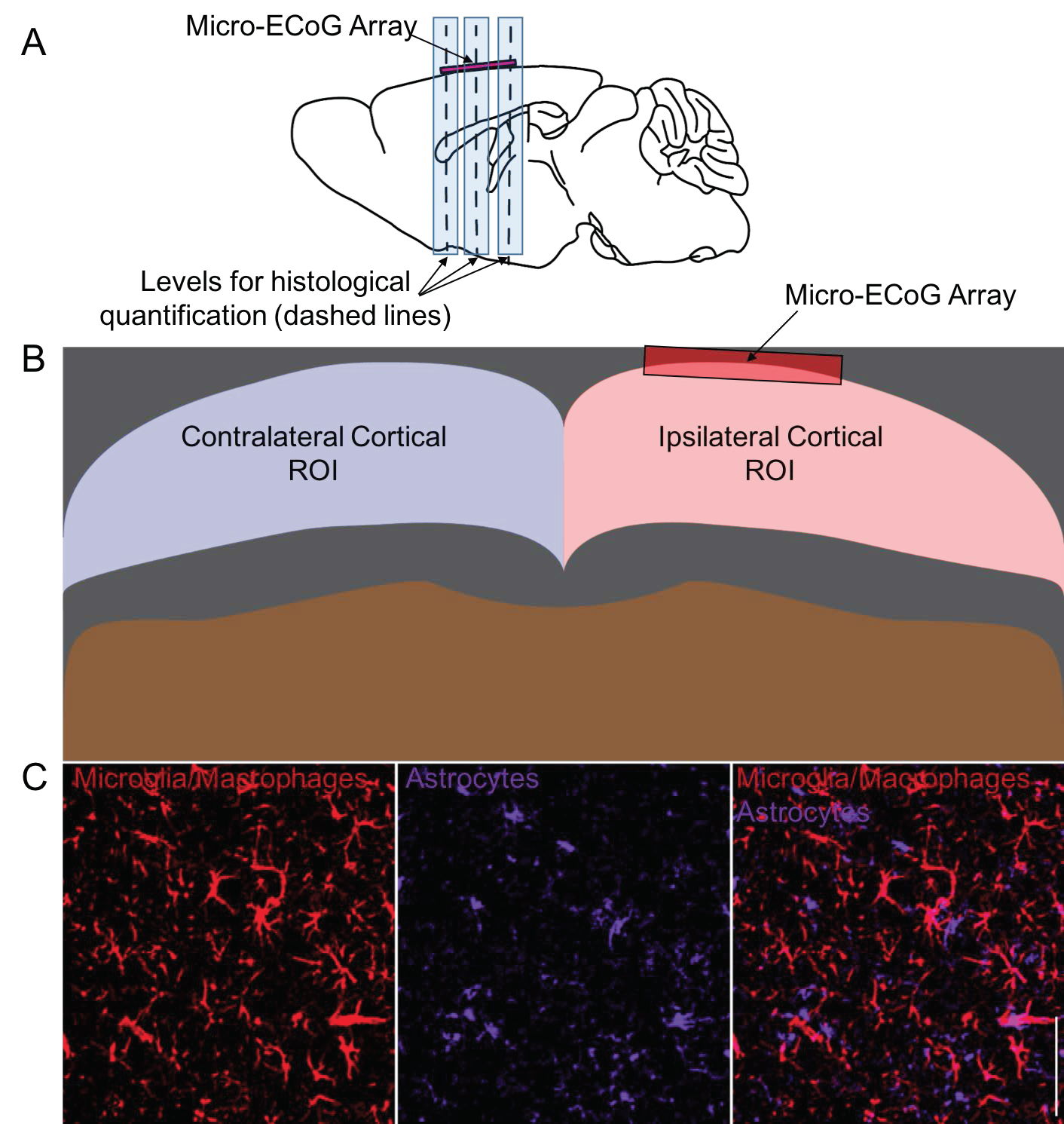

Supplement: S1 Fig — (A) After perfusion, the microECoG arrays were carefully removed and brains were divided into three tissue blocks containing the regions contacting the micro-ECoG array. Blocks were cryoprotected in 30% sucrose and frozen. Sections were then serially cut 20 μm thick from the three levels and stained for Iba1 (microglia/macrophages) and GFAP (astrocytes). (B) Glial reactivity was quantified in an automated fashion by measuring the pixel intensity of the ipsilateral cortex ROI normalized to the contralateral cortical ROI (10x objective, 1024x1024). (C) To quantify the number of microglia/macrophage, representative regions from the ipsilateral cortices below the arrays were imaged with a 20x objective (area: 1024x1024 pixels, 1100 μm x 881 μm), and Iba1+ cells were manually counted. Scale bar: 100 μm. (TIFF) [file pone.0206137.s002.tiff]

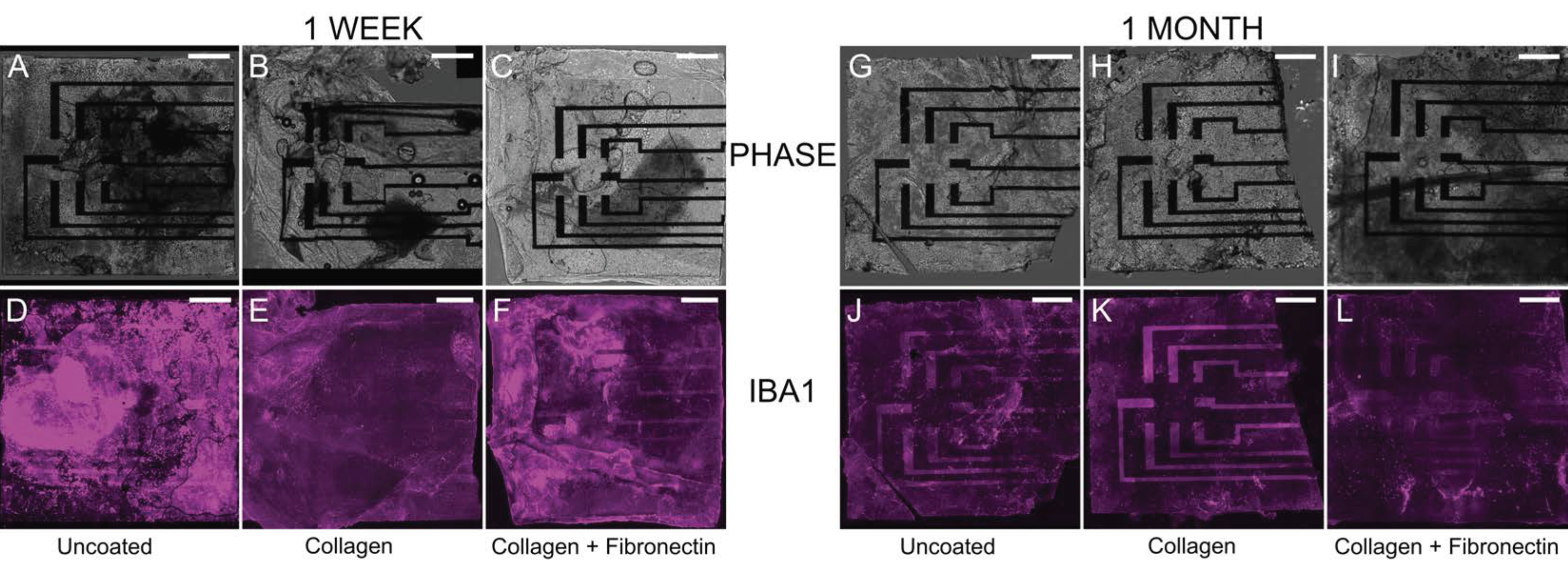

Supplement: S2 Fig — Representative phase images (A-C, G-I) and multiphoton reconstructions (D-F, J-L) of uncoated (A/D, G/J), collagen-coated (B/E, H/K), and fibronectin-coated (C/F, I/L) microECoG arrays at 1 week (A-F) and 1 month (G-L) post-implant. Arrays were immunolabeled for IBA-1 to identify activated microglia/macrophages. Scale bars: 500 μm. (TIFF) [file pone.0206137.s003.tiff]
